# Teenage pregnancy as a risk factor for placental abruption: Findings from the prospective Japan environment and children’s study

**DOI:** 10.1371/journal.pone.0251428

**Published:** 2021-05-13

**Authors:** Hyo Kyozuka, Tsuyoshi Murata, Toma Fukusda, Akiko Yamaguchi, Aya Kanno, Shun Yasuda, Akiko Sato, Yuka Ogata, Yuta Endo, Mitsuaki Hosoya, Seiji Yasumura, Koichi Hashimoto, Hidekazu Nishigori, Keiya Fujimori, the Japan Environment and Children’s Study (JECS) Group

**Affiliations:** 1 Fukushima Regional Center for the Japan Environmental and Children’s Study, Fukushima, Japan; 2 Department of Obstetrics and Gynecology, Fukushima Medical University School of Medicine, Fukushima, Japan; 3 Department of Pediatrics, Fukushima Medical University School of Medicine, Fukushima, Japan; 4 Department of Public Health, Fukushima Medical University School of Medicine, Fukushima, Japan; 5 Fukushima Medical Center for Children and Women, Fukushima Medical University School of Medicine, Fukushima, Japan; University of Mississippi Medical Center, UNITED STATES

## Abstract

**Objective:**

Placental abruption is a significant obstetric complication that affects both maternal and neonatal mortality and morbidity. The present study examined the effect of maternal age on the incidence of placental abruption.

**Methods:**

We used data of singleton pregnancies from the Japan Environment and Children’s Study, which was a prospective birth cohort study conducted between January 2011 and March 2014 across 15 regional centers in Japan. A multiple regression model was used to identify whether maternal age (<20 years, 20–24 years, 25–29 years, 30–34 years, and ≥35 years) is a risk factor for placental abruption. The analyses were conducted while considering the history of placental abruption, assisted reproductive technology, number of previous deliveries, smoking during pregnancy, body mass index before pregnancy, and chronic hypertension.

**Results:**

A total of 94,410 Japanese women (93,994 without placental abruption and 416 with placental abruption) were recruited. Herein, 764, 8421, 25915, 33517, and 25793 women were aged <20 years, 20–24 years, 25–29 years, 30–34 years, and ≥35 years, respectively. Besides advanced maternal age (≥35 years; adjusted odds ratio: 1.7, 95% confidence interval: 1.1–2.5), teenage pregnancy was also a risk factor for placental abruption (adjusted odds ratio: 2.8, 95% confidence interval: 1.2–6.5) when the maternal age of 20–24 years was set as a reference.

**Conclusions:**

In the Japanese general population, besides advanced maternal age, teenage pregnancy was associated with placental abruption. Recently, the mean maternal age has been changing in Japan. Therefore, it is important for obstetric care providers to provide proper counseling to young women based on up-to-date evidence.

## Introduction

Placental abruption is a significant obstetric complication that affects both maternal and neonate mortality and morbidity. It is defined as the premature partial or total separation of a normally implanted placenta [1]. Maternal consequences include excessive blood loss and disseminated intravascular coagulation, sometimes requiring a blood transfusion; it can lead to hypovolemic shock, multiorgan failure, peripartum hysterectomy, and rarely, death [2, 3]. Neonatal consequences include preterm birth (PTB) and related hypoxia or asphyxia [1, 4]. Fetal asphyxia combined with prematurity can be associated with short-term sequelae such as neonatal encephalopathy/hypoxic-ischemic encephalopathy and long-term sequelae such as cerebral palsy, lung diseases, and epilepsy [1, 5].

Several risk factors, such as advanced and younger maternal age; number of previous deliveries; hypertension before pregnancy; maternal smoking; ethnicity [2, 6–8]; and pregnancy-related complications such as preeclampsia, chorioamnionitis, and preterm rupture of membrane, a complication resulting in preterm birth [9], have been reported for placental abruption.

The prevalence of placental abruption varies across regions. In some countries, the prevalence has been increasing since the past decade, possibly due to changes in the risk factors [2], including increasing maternal age, body mass index, and increasing use of assisted reproductive technology (ART). The statistics suggest that the mean maternal age is increasing, indicating that maternal age could be a key risk factor for obstetrics complications in Japan [10]. Besides, as we have reported earlier, teenage maternal age is also a risk factor for severe maternal obstetric complications such as hypertensive disorders of pregnancy and placental abruption in Fukushima prefecture, Japan [11]. Owing to social changes in the last decade, such as an increase in mean maternal age at delivery, up-to-date evidence for the effect of maternal age on placental abruption is required.

Several studies have focused on advanced maternal age as a risk factor for placental abruption. However, a few prospective studies have comprehensively assessed both teenage and advanced maternal age as risk factors for placental abruption, while accounting for several confounding factors by including a large number of participants of the same ethnicity. Therefore, here, we evaluated the effect of maternal age on placental abruption; the participants were enrolled from the largest prospective birth cohort study of Japan, conducted between 2011 and 2014.

## Materials and methods

In this study, the data from Japan Environment and Children’s Study (JECS), a nationwide, government-funded, birth cohort study evaluating the effects of environmental factors on children’s health [12] that began in January 2011, was investigated. The eligibility criteria for the participants (expectant mothers) were as follows: (1) residing in one of the study areas at the time of recruitment and expected to reside continually in Japan for the foreseeable future, (2) an expected delivery date between August 01, 2011 and mid-2014, and (3) the ability to participate in the study without difficulty (i.e., the participant needed to be able to comprehend the Japanese language and complete the self-administered questionnaires). This study was conducted at 15 regional centers across Japan; the details have been described elsewhere [12].

### Data collection

The current analysis used the JECS dataset released in June 2016 (dataset: jecs-ag-20160424). Two types of data were used: (1) T1, the data regarding basic maternal characteristics and obstetric history. It was obtained from the self-reported questionnaires collected during the first trimester (the first questionnaire); and (2) M0, the data regarding the maternal medical background and obstetrical outcomes. These were retrieved from the medical records provided by health care providers. The participants with multiple gestation pregnancies and those with missing data were excluded from the analysis. None of the cases was delivered at <22 weeks of gestation.

### Maternal information

The maternal information was obtained from both M0 and T1 data. The M0 data included information on maternal age at the time of delivery, pre-pregnancy body mass index, presence of maternal chronic hypertension, and parity. The T1 data included information on previous pregnancy complications, maternal smoking status, and manner of conception. The method of conception was categorized as natural or ART-related, with ART defined as conception after in vitro fertilization and/or intracytoplasmic sperm injection, or cryopreserved, frozen, blastocyst or embryo transfers [13]. The mothers were categorized into the following five groups based on their age: <20 years, 20–24 years, 25–29 years, 30–34 years, and ≥35 years. The mothers were also stratified based on the number of previous deliveries—0, 1, 2, 3, and ≥4. Body mass index was calculated according to the criterion of the World Health Organization (body weight [kg]/height^2^ [m^2^]). We further categorized the participants into three groups according to their body mass index: <18.5 kg/m^2^, 18.5–25.0 kg/m^2^, and ≥25.0 kg/m^2^. A self-report questionnaire during the first trimester provided information on the participants’ smoking habit—“Never,” “Previously did, but quit before realizing current pregnancy,” and “Currently smoking.” Women in the “Currently smoking” category were considered smokers, while the others were considered non-smokers. Maternal chronic hypertension was defined as the presence of hypertension (systolic blood pressure >140 mmHg or diastolic blood pressure >90 mmHg) before conception. The process of collecting data for pre-pregnancy gynecological complications from the self-report questionnaire of JECS has been validated previously [14, 15].

### Obstetrical outcomes

The data for obstetrical outcomes were obtained from the M0 data and included the following: gestational age at the time of delivery, the presence or absence of placental abruption, mode of delivery, umbilical artery (UmA) pH, intrauterine fetal death (IUFD) and maternal transfusion. The mode of delivery was categorized into vaginal delivery or cesarean section (CS). In the present study, placental abruption was diagnosed clinically based on the clinical findings of abdominal pain, vaginal bleeding, uterine contractions, fetal distress, and vital signs’ abnormalities at the discretion of the obstetrician-in-charge. Histological confirmation was not mandatory for the diagnosis of placental abruption during the present analysis. Fetal arterial blood was obtained at the time of the delivery, and UmA pH was measured immediately after delivery. Fetal acidosis was stratified by UmA pH (<7.20, <7.10, and <7.00) based on a previous study, according to which a pH of 7.20 was associated with an increased risk of adverse short-term outcomes [16], pH of 7.10 was associated with an increased risk of adverse neurological sequelae [17], and pH <7.00 was more frequently associated with cerebral palsy [18]. IUFD was defined as the loss of fetal heartbeat after 22 weeks of gestation.

### Statistical analyses

The data of the women with placental abruption were reviewed. The maternal background and obstetric outcomes were compared between women with and without placental abruption. The frequency of placental abruption was also examined according to the gestational age. The chi-square test and Fisher’s exact test were used to compare the categorical variables, and a t-test was used to compare the continuous variables after confirming that each of the continuous variables was normally distributed. The adjusted OR (aOR) and 95% CI for placental abruption were calculated using the multiple logistic regression model. The ORs were adjusted for confounding variables, including the history of placental abruption, ART pregnancy, parity, maternal age, smoking during pregnancy, body mass index before pregnancy, and chronic hypertension. In the logistic regression model, dummy variables were used for the categorical variables that consisted of more than three categories. All statistical analyses were conducted using SPSS, version 26 (IBM Corp., Armonk, NY, USA). The differences with *P*<0.05 were considered statistically significant.

### Ethical approval

The JECS protocol was reviewed and approved by the Ministry of the Environment’s Institutional Review Board for Epidemiological Studies (No.100910001). The JECS was conducted in accordance with the principles of the Declaration of Helsinki and other valid national regulations and guidelines. Written informed consent was obtained from all participating women. There was no patient and public involvement in this study. We analyzed fully anonymized data-set during April 2020 and August 2020.

## Results

**Fig 1:**
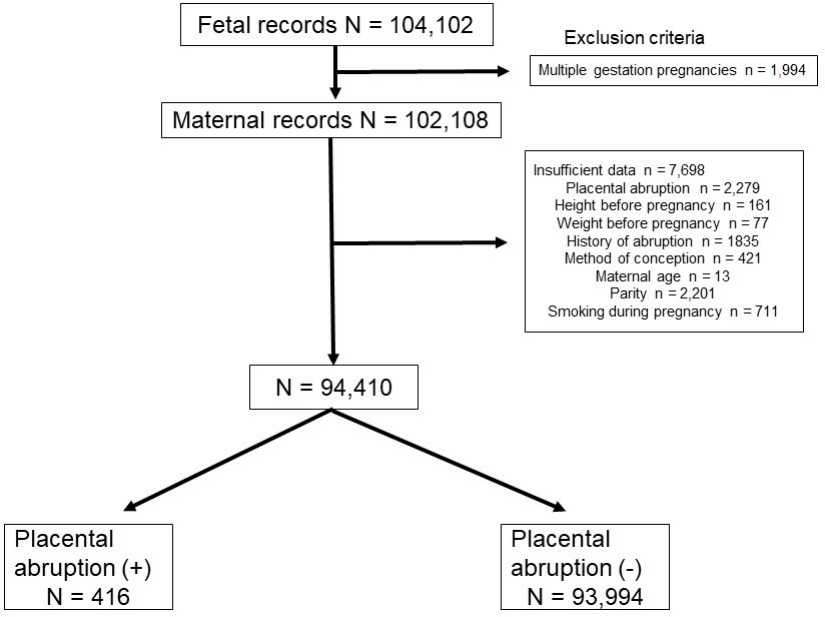
Flowchart for enrolment and inclusion of participants in the analysis.

**Fig 2:**
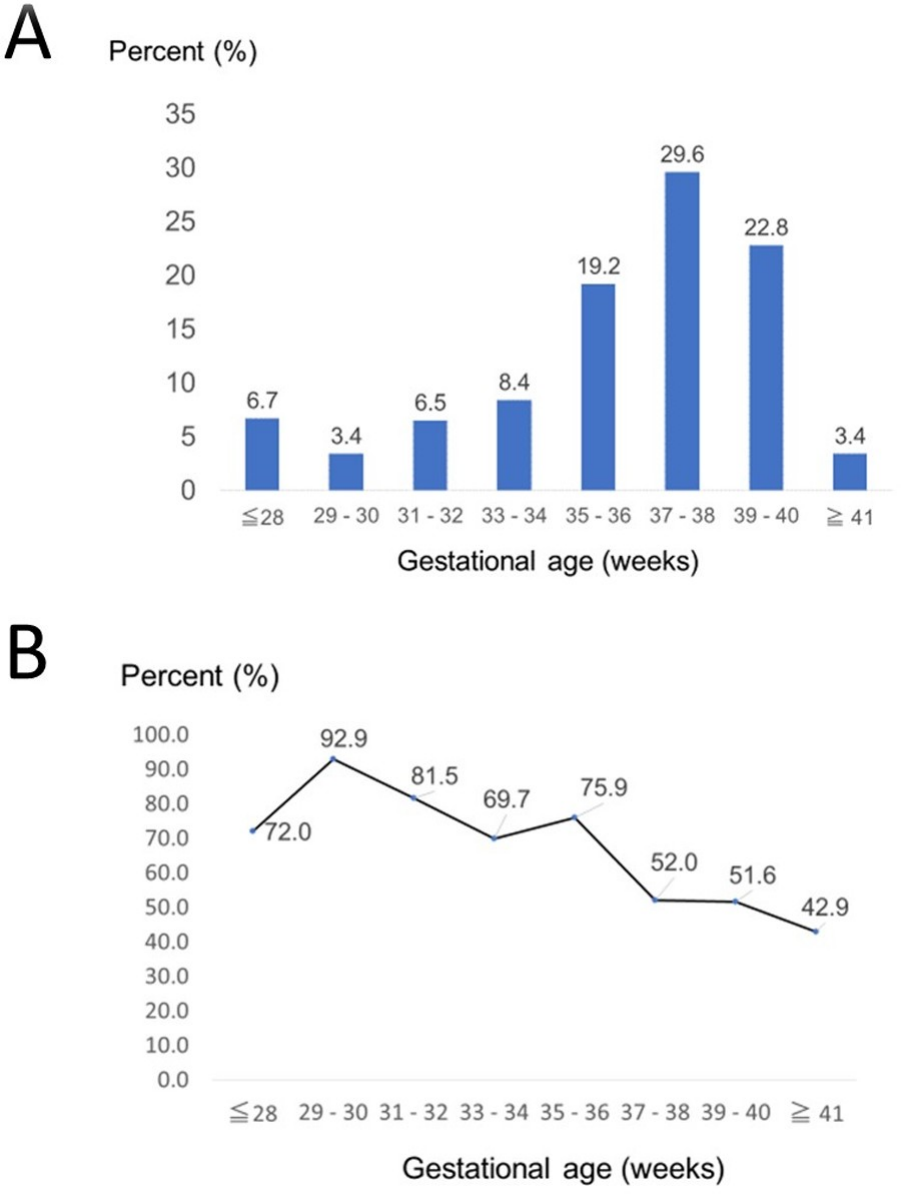
**(A)** The prevalence of placental abruption stratified by gestational age. **(B)** The rate of cesarean section among women with placental abruption stratified by gestational age.

In total, 104,102 women were identified during the study period. Of these, 1,994 women who had multiple gestation pregnancies and 7,698 women, whose data were missing, were excluded from the analysis (Fig 1). After applying our exclusion criteria, the data of 94,410 maternal participants were included in the analysis. Among 94,410 participants, 416 women had a placental abruption and 93,994 women did not have placental abruption. The prevalence rate of placental abruption was 0.4% (416/94,410). Almost half (44.1%) of the placental abruption cases occurred prematurely before the gestational age of 37 weeks (Fig 2A).

Table 1 summarizes the maternal characteristics and obstetrical outcomes of the participants stratified by the presence and absence of placental abruption.

**Table 1:** Basic characteristics of the participants stratified by the presence and absence of placental abruption.

Variable	Placental abruption (+) N = 416	Placental abruption (-) N = 93,994	*P* value
Maternal age (years), mean (SD)	32.0 (5.2)	31.2 (5.0)	0.003^a^
Maternal age category, %			
≦19 years	1.7	0.8	0.001^b^
20–24 years	6.5	8.9	
25–29 years	20.9	27.5	
30–34 years	37.3	35.5	
≧35 years	33.7	27.3	
Parity, %			
0	40.9	40.2	0.458^b^
1	35.5	39.1	
2	19.0	16.3	
3	3.6	3.5	
≥ 4	1.2	1.0	
History of placental abruption, %	1.0	0.2	0.021^c^
ART pregnancy, %	5.5	2.9	0.002^b^
Smoking during pregnancy, %	7.9	4.9	0.004^b^
Chronic hypertension, %	5.3	1.3	< 0.001^b^
BMI, %	9.5	0.4	<0.001^b^
< 18.5 kg/m^2^	17.3	16.1	0.144^b^
18.5–24.9 kg/m^2^	69.3	73.1	
≥ 25 kg/m^2^	13.4	10.8	
Obstetrical outcomes			
Cesarean section, %	62.2	18.6	< 0.001^b^
UmA pH, mean (SD)	7.21 (0.18)	7.32 (0.12)	< 0.001^a^
UmA pH < 7.20, %	30.7	6.2	< 0.001^b^
UmA pH < 7.10, %	16.1	1.1	< 0.001^b^
UmA pH < 7.00, %	10.9	0.2	< 0.001^b^
IUFD, %	9.5	0.4	<0.001^b^
Maternal blood transfusion, %	1.7	0.5	0.004^c^

**Abbreviations:** SD: Standard deviation; ART: Assisted reproductive technology; BMI: Body mass index; UmA: Umbilical artery; IUFD: Intrauterine fetal death.

^a^ *P*-value from t-test.

^b^ *P*-value from Chi-square test.

^c^ *P-* value from Fisher’s exact test.

*P*<0.05 indicates statistical significance.

The rate of CS in women with placental abruption was 62.2%, which was significantly higher than that in women without placental abruption (18.6%, *P*<0.001). Among women with placental abruption, the rate of CS dropped rapidly after 37 weeks of pregnancy (Fig 2B); the CS rates before and after 37 weeks of gestation among women with placental abruption were 76.8% and 51.3%, respectively (p<0.001)

Table 2 shows the results of the logistic regression analyses. After adjusting for potential confounding factors, the history of placental abruption (adjusted odds ratio (aOR): 3.5, 95% CI: 1.3–9.6, *P* = 0.014), ART (aOR: 1.7, 95% CI: 1.1–2.7, *P* = 0.024), maternal age < 20 years (aOR: 2.8, 95% CI: 1.2–6.5, *P* = 0.016), maternal age ≥ 35 years (aOR: 1.7, 95% CI: 1.1–2.5, *P* = 0.028), smoking during pregnancy (aOR: 1.7, 95% CI: 1.2–2.5, *P* = 0.003), and chronic hypertension before pregnancy (aOR: 4.0, 95% CI: 2.5–6.2, *P*<0.001) were associated with placental abruption. When no parity was set as a reference, no association was observed between the number of parities and the risk of placental abruption.

**Table 2:** Factors associated with placental abruption: Results from univariate and multivariate logistic regression analyses.

Variable	Univariate analysis			Multivariate analysis		
	OR	95% CI	*P* value	aOR	95% CI	*P* value
History of placental abruption	3.9	1.5–10.7	0.007	3.5	1.3–9.6	0.014
ART pregnancy	2.0	1.3–3.0	0.002	1.7	1.1–2.7	0.024
Parity						
0	Ref			Ref		
1	0.9	0.7–1.1	0.303	0.9	0.7–1.1	0.246
2	1.1	0.9–1.5	0.315	1.1	0.8–1.4	0.647
3	1.0	0.6–1.7	0.956	0.9	0.5–1.5	0.637
≥ 4	1.2	0.5–3.0	0.648	1.0	0.4–2.4	0.975
Maternal age, years						
< 20	2.9	1.2–6.6	0.013	2.8	1.2–6.5	0.016
20–24	Ref			Ref		
25–29	1.0	0.7–1.6	0.834	1.1	0.7–1.6	0.810
30–34	1.4	1.0–2.2	0.078	1.5	1.0–2.2	0.075
≧35	1.7	1.1–2.6	0.012	1.7	1.2–2.5	0.028
Smoking during pregnancy	1.7	1.2–2.4	0.004	1.7	1.2–2.5	0.003
BMI						
< 18.5 kg/m^2^	1.1	0.9–1.5	0.346	1.2	0.9–1.5	0.243
18.5–24.9 kg/m^2^	Ref			Ref		
≥ 25 kg/m^2^	1.3	1.0–1.8	0.062	1.1	0.8–1.5	0.395
Chronic hypertension	4.3	2.8–6.7	< 0.001	4.0	2.5–6.2	< 0.001

**Abbreviations:** OR: Odds ratio; CI: Confidence interval; aOR: Adjusted odds ratio; ART: Assisted reproductive technology; Ref: Reference; BMI: Body mass index.

## Discussion

In this study, we examined the risk factors for placental abruption based on the data of a large cohort study conducted in Japan. Consistent with several studies, the present study demonstrated that the history of placental abruption, ART pregnancy, smoking during pregnancy, chronic hypertension, and maternal age were the risk factors for placental abruption. Compared to advanced maternal age, teenage maternal age was more strongly associated with placental abruption. The number of previous deliveries was not associated with the incidence of placental abruption.

The prevalence of placental abruption varies across regions. In the present study, the incidence of placental abruption was 0.4%, which is similar to that reported in the Nordic countries (0.4–0.5%) and lower than that reported in the US (0.6–1.0%) [2]. Postpartum hemorrhage is the most frequently reported maternal morbidity associated with placental abruption, and as a consequence of placental abruption, postpartum hemorrhage increases the incidence of maternal blood transfusion. The rate of transfusion due to placental abruption in the present study was 1.7%, which is lower than that reported in the previous studies (2.4–14.6%) [1]. The distinctive difference in maternal transfusion rate for women with placental abruption may be due to different diagnostic criteria for placental abruption in each study. The diagnosis of abruption is primarily clinical, but sometimes findings from the imaging, laboratory, and postpartum pathologic studies can be used to support the clinical diagnosis [1]. However, to date, there is no gold standard for diagnosing placental abruption.

The findings of the present study indicate that half of the placental abruption cases occurred before 37 weeks of gestation. PTB is one of the most frequently reported obstetrical outcomes associated with placental abruption [1]. PTB has two clinical subtypes, viz., spontaneous PTB and medically indicated PTB; the former occurs due to preterm rupture of membrane and chorioamnionitis, the latter occurs due to hypertensive disorders of pregnancy [9, 19]. Placental abruption can lead to both spontaneous and medically indicated PTB. Spontaneous PTB due to placental abruption is thought to be the result of bleeding from the separation of the placenta, which irritates the uterine lining and stimulates uterine contractions, leading to PTB [20]. Medically indicated PTB, because of placental abruption, is usually performed by CS to reduce the risk of maternal and perinatal morbidity and mortality [21]. The present study showed a high prevalence of CS (62.2%) in women with placental abruption and a significantly high percentage of CS before 37 weeks of gestation (Fig 2B), suggesting that most cases of PTB were medically indicated.

Placental abruption is a complex outcome of pregnancy. Although several risk factors for placental abruption are known, its etiopathogenesis is not fully understood. Ananth et al. reported that instead of the number of previous deliveries, maternal age was an independent risk factor for placental abruption [22]. The findings of the present study are consistent with those reported by Ananth et al. The underlying reason why advanced maternal age increases the risk of placental abruption is speculative. Most abruptions appear to be related to a chronic placental disease process, wherein, abnormalities in the early development of the spiral arteries, which could be affected by maternal age, can lead to decidual necrosis, placental inflammation, and possibly infarction, ultimately resulting in vascular disruption and bleeding [23–25].

A few studies have reported the association between teenage pregnancy and placental abruption. Our former preliminary report with descriptive analysis reported that maternal age <20 years was associated with the highest occurrence of placental abruption in Fukushima prefecture Japan [11]. We concluded that low socio-economic status could be associated with the high occurrence of severe maternal complications among teenagers. This study shows that maternal age <20 years was a risk factor for placenta abruption despite taking into account factors such as maternal smoking status, which is one of the factors representing the socio-economic status. The underlying reason why teenage pregnancy is an independent risk factor for placental abruption in the present analysis is speculative. However, the findings of the present study must be interpreted with caution because teenage women are likely to be associated with less education, low income, and malnutrition, which have not been considered as confounding factors in the present analysis. Placental abruption during teenage could be due to direct mechanical events such as blunt abdominal trauma and/or rapid uterine decompression, the occurrence of which are more likely in young maternal age [26].

The strength of the present study is that it is the first large-scale, nationwide, birth cohort study investigating various factors contributing to placental abruption in pregnant women of Japan. Therefore, the findings of this study can be considered to be representative of not only Fukushima prefecture where catastrophic natural and environmental disaster occurred but also the general pregnant population of Japan [27]. The prospective data were collected by the physicians, midwives, nurses, and trained research coordinators and, therefore, are more likely to be accurate.

This study also has a few limitations. First, this study lacked the definition of placental abruption and the data for type (occurred during antepartum or intrapartum) and severity. The severity of placental abruption could have been graded based on the maternal (disseminated intravascular coagulation, hypovolemic shock, renal failure), fetal (non-reassuring fetal status, intrauterine fetal growth restriction, intrauterine fetal death), or neonatal (preterm delivery, small for gestational age, or neonatal death) complications [28]. The severity usually causes premature placental separation. Therefore, the higher occurrence of abruption-related complications such as the rate of CS; pH<7.20, <7.10, and <7.00; IUFD; and maternal transfusion could minimize this limitation. Second, the specific ART methods (in vitro fertilization and/or intracytoplasmic sperm injection; cryopreserved, frozen, or blastocyst embryo transfer) were not classified in this study. Third, this study included individuals of the same ethnicity, therefore whether these results can be applied to other ethnic groups is unknown.

Finally, although several confounding factors were accounted for based on the questionnaire, unknown risk factors for placental abruption might have existed. Further studies are warranted to elucidate the potential impact of these confounding factors on placental abruption and how these factors can impact the clinical practice of all obstetric care providers.

Conventionally, aging is thought to be a risk factor for placental abruption. Therefore, this study focused on advanced maternal age for accessing maternal obstetric complications. The findings of the present study suggest that young maternal age is more significantly associated with placental abruption than the advanced maternal age. Owing to the social changes in the last decade, an increase in mean maternal age has been observed, along with the advances in ART, and an increasing number of women pursuing higher education and careers [19]; therefore, it is important for obstetric care providers to provide proper counseling to young women based on the up-to-date evidence.
